# Time-kill curve analysis and pharmacodynamic modelling for in vitro evaluation of antimicrobials against *Neisseria gonorrhoeae*

**DOI:** 10.1186/s12866-016-0838-9

**Published:** 2016-09-17

**Authors:** Sunniva Foerster, Magnus Unemo, Lucy J. Hathaway, Nicola Low, Christian L. Althaus

**Affiliations:** 1 Institute of Social and Preventive Medicine (ISPM), University of Bern, Bern, Switzerland; 2 Institute for Infectious Diseases, University of Bern, Bern, Switzerland; 3 WHO Collaborating Centre for Gonorrhoea and other STIs, National Reference Laboratory for Pathogenic Neisseria, Faculty of Medicine and Health, Örebro University, Örebro, Sweden; 4 Graduate School for Cellular and Biomedical Sciences, University of Bern, Bern, Switzerland

**Keywords:** *Neisseria gonorrhoeae*, Gonorrhoea, Antimicrobial resistance, Time-kill curves, Pharmacodynamics

## Abstract

**Background:**

Gonorrhoea is a sexually transmitted infection caused by the Gram-negative bacterium *Neisseria gonorrhoeae*. Resistance to first-line empirical monotherapy has emerged, so robust methods are needed to evaluate the activity of existing and novel antimicrobials against the bacterium. Pharmacodynamic models describing the relationship between the concentration of antimicrobials and the minimum growth rate of the bacteria provide more detailed information than the MIC only.

**Results:**

In this study, a novel standardised in vitro time-kill curve assay was developed. The assay was validated using five World Health Organization *N. gonorrhoeae* reference strains and a range of ciprofloxacin concentrations below and above the MIC. Then the activity of nine antimicrobials with different target mechanisms was examined against a highly antimicrobial susceptible clinical strain isolated in 1964. The experimental time-kill curves were analysed and quantified with a previously established pharmacodynamic model. First, the bacterial growth rates at each antimicrobial concentration were estimated with linear regression. Second, we fitted the model to the growth rates, resulting in four parameters that describe the pharmacodynamic properties of each antimicrobial. A gradual decrease of bactericidal effects from ciprofloxacin to spectinomycin and gentamicin was found. The beta-lactams ceftriaxone, cefixime and benzylpenicillin showed bactericidal and time-dependent properties. Chloramphenicol and tetracycline were purely bacteriostatic as they fully inhibited the growth but did not kill the bacteria. We also tested ciprofloxacin resistant strains and found higher pharmacodynamic MICs (zMIC) in the resistant strains and attenuated bactericidal effects at concentrations above the zMIC.

**Conclusions:**

*N. gonorrhoeae* time-kill curve experiments analysed with a pharmacodynamic model have potential for in vitro evaluation of new and existing antimicrobials. The pharmacodynamic parameters based on a wide range of concentrations below and above the MIC provide information that could support improving future dosing strategies to treat gonorrhoea.

**Electronic supplementary material:**

The online version of this article (doi:10.1186/s12866-016-0838-9) contains supplementary material, which is available to authorized users.

## Background

Antimicrobial resistance in *Neisseria gonorrhoeae* is a major public health problem. Strains that have developed resistance to all antimicrobials used for treatment have been classified as superbugs [1–3]. Clinical resistance to the last option for empirical antimicrobial monotherapy, ceftriaxone, was first described in 2009 [4]. Currently, treatment recommendations for gonorrhoea and prediction of the efficacy of antimicrobials mainly rely on a single measurement: the MIC of the antimicrobial, sometimes supported by data from old clinical trials and pharmacokinetic/pharmacodynamic (PK/PD) simulations. However, antimicrobials that have different modes of action and lead to different treatment outcomes can have identical MICs [5]. A better understanding of the in vitro pharmacodynamic properties of antimicrobials could be used to optimise dosing strategies and help prevent treatment failures [6].

**Fig. 1:**
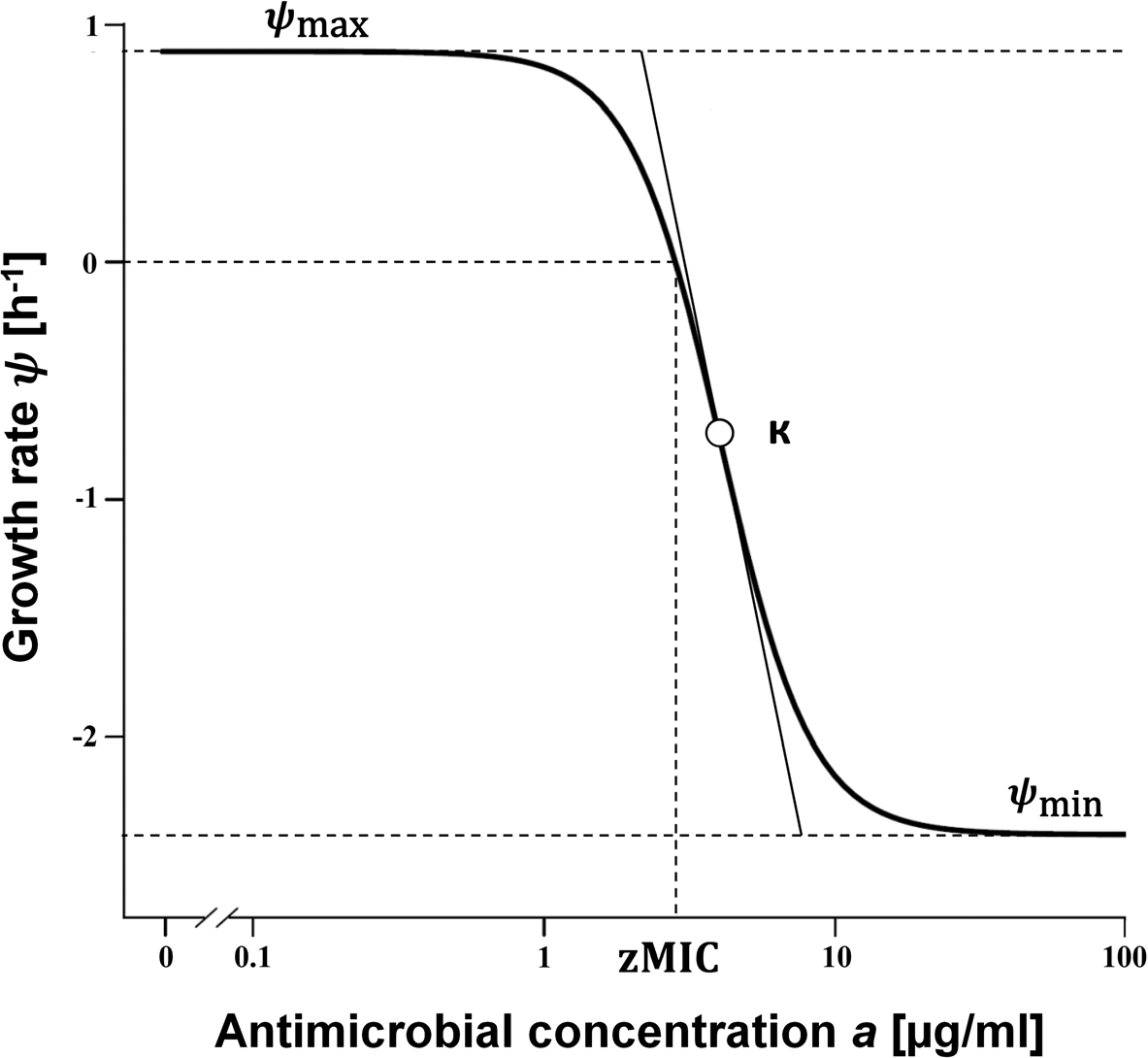
Pharmacodynamic model with four parameters. The bacterial growth rates (*ψ*) in response to each antimicrobial concentration are estimated from time-kill data with linear regression. The maximal bacterial growth rate *ψ*_max_, the minimal bacterial growth rate at high concentrations of antimicrobial *ψ*_min_, the pharmacodynamic MIC (zMIC) and the Hill coefficient *к* are shown and define the shape of the curve

Time-kill curves that monitor bacterial growth and death over a wide range of antimicrobial concentrations have been frequently used to evaluate the effect of antimicrobials over time. These data can be analysed using mathematical models and are often the first step in PK/PD modelling. Regoes et al. [7] analysed time-kill curves from *E. coli* exposed to different classes of antimicrobials using a pharmacodynamic model that is characterised by four parameters: the maximal bacterial growth rate in the absence of antimicrobial (*ψ*_max_), the minimal bacterial growth rate at high concentrations of antimicrobial (*ψ*_min_), the Hill coefficient (*к*), and the pharmacodynamic MIC (zMIC) (Fig. 1). This model, which is closely related to *E*_max_ models [5], has also been applied to study the effects of antibiotics alone and in combinations against other pathogens, such as *Staphylococcus aureus* [8] and *Mycobacterium marinum* [9].

Information about the effects of antimicrobials covering a wide range of antimicrobial concentrations below and above the MIC is particularly valuable for pathogens like *N. gonorrhoeae*, because data about PK/PD effects are limited. There is no standardised and quality assured time-kill curve analysis or animal model for the fastidious obligate human pathogen *N. gonorrhoeae*. Most published time-kill protocols for *N. gonorrhoeae* [10–12] are not generalizable, owing to the highly divergent growth requirements of different strains and interpretation of results generally relies on qualitative expert judgement. To study a wide range of *N. gonorrhoeae* strains, growth in absence of antimicrobials must be consistent and bacterial growth phases at the time of exposure to antimicrobial need to be synchronised in early to mid-log phase.

In this study, a standardised in vitro time-kill curve assay for *N. gonorrhoeae* was developed using Graver-Wade (GW) medium. GW medium is a chemically defined, nutritious, liquid medium that supports growth of a wide range of *N. gonorrhoeae* auxotypes and clinical isolates starting from very low inocula [13]. The novel time-kill curve assay was validated on five World Health Organization *N. gonorrhoeae* reference strains with fluoroquinolone resistance determinants. A highly susceptible clinical *N. gonorrhoeae* isolate (DG666, isolated in 1964) was subsequently studied in detail and time-kill curve experiments performed for nine antimicrobials that have been, or currently are, used to treat gonorrhoea. In a second step we analysed the time-kill data using a pharmacodynamic model [7] for a comparative analysis of the pharmacodynamic properties of different antimicrobials.

## Methods

### *Neisseria gonorrhoeae* isolates and media

The five international *N. gonorrhoeae* reference strains WHO G, WHO K, WHO L, WHO M, and WHO N with different ciprofloxacin conferring mutations in *gyrA*, *parC* and *parE* [14, 15] and a clinical isolate susceptible to all antimicrobials that were examined (wild type) cultured in 1964 (DG666), were studied. Isolates were cultured, from frozen stocks (−70 °C), on GCAGP agar plates (3.6 % Difco GC Medium Base agar [BD, Diagnostics, Sparks, MD, USA] supplemented with 1 % haemoglobin [BD, Diagnostics], 1 % IsoVitalex [BD, Diagnostics] and 10 % horse serum) for 18–20 h at 37 °C in a humid 5 % CO_2_-enriched atmosphere. Gonococcal colonies were subcultured once more on GCAGP agar for 18–20 h at 37 °C in a humid 5 % CO_2_-enriched atmosphere, before being transferred to the liquid sterile GW medium, prepared as earlier described [13], for growth curve and time-kill experiments.

### Viable cell counts

Bacterial viability was measured using a modified Miles and Misra method as previously described [16]. Growing bacteria were removed from 96-well plates at specified time points using a multichannel pipette and diluted in sterile phosphate buffered saline (PBS) in six subsequent 1:10 dilutions (20 μl culture in 180 μl diluent). Ten μl droplets of each dilution were spotted on GCRAP (3.6 % Difco GC Medium Base agar [BD, Diagnostics] supplemented with 1 % haemoglobin [BD, Diagnostics] and 1 % IsoVitalex [BD, Diagnostics]). GCRAP plates were dried with the lid open in a sterile environment for 30–60 min before use. After drying the droplets (approximately 5–10 min), plates were incubated for 24 h at 37 °C in a humid 5 % CO_2_-enriched atmosphere. For every concentration and time point, colonies were counted for the first dilution that resulted in a countable range of 3–30 colonies and the CFU/ml calculated.

### Growth curves

Prior to growth curve experiments, strains were subcultured once on chocolate agar PolyViteX (Biomerieux). A 0.5 McFarland inoculum was prepared and diluted to 100 CFU/ml (1:10^6^) in GW Medium (35 °C). A volume of 100 μl diluted bacteria per well was transferred to Sarstedt round-bottom 96 well plates. The plates were tightly sealed with adhesive polyester foil (Sarstedt) and bacteria were grown shaking at 100 rpm at 35 °C in a humid 5 % CO_2_-enriched atmosphere. Bacterial growth was monitored over a time-course of 60 h (0, 2, 4, 6, 8, 10, 12, 20, 22, 24, 26, 28, 30, 32, 34, 40, 44, 48, 60 h). For every sampled time point, the content of one well was removed and viable counts determined [16]. Growth curves were analysed by plotting the log CFU/ml against the time and fitting a Gompertz growth model [17] to the data as implemented in the package *cellGrowth* [18] for the R software environment for statistical computing [19]. Only lag, log and stationary phases were included in the analysis and the decline phase excluded.

### Time-kill assay

Time-kill curve analyses were performed by culturing *N. gonorrhoeae* in GW medium [13], in the presence of 11 antimicrobial concentrations in doubling dilutions ranging from 0.016 × MIC to 16 × MIC. For DG666, the MICs were determined before the experiment using Etest (bioMérieux, Marcy l’Etoile, France) according to the manufacturer’s instructions. For all other strains, previously published MIC values were used [14]. The antimicrobials examined were ciprofloxacin (Sigma Aldrich, China), gentamicin (Sigma Aldrich, Israel), spectinomycin (Sigma Aldrich, Israel), azithromycin (Sigma Aldrich, USA), benzylpenicillin (Sigma Aldrich, USA), ceftriaxone (Sigma Aldrich, Israel), cefixime (European pharmacopeia reference standard, France), chloramphenicol (Sigma Aldrich, China) and tetracycline (Sigma Aldrich, China). Growth curves were initially performed to confirm that all strains would reach a stable early- to mid-log phase after 4 h of pre-incubation in antimicrobial-free GW medium. A 0.5 McFarland inoculum of *N. gonorrhoeae* was then prepared in sterile PBS from cultures grown on GCAGP agar plates for 18–20 h at 37 °C in a humid 5 % CO_2_-enriched atmosphere. For each strain, 30 μl of the inoculum was diluted in 15 ml pre-warmed (37 °C) antimicrobial-free GW medium and 90 μl per well was dispersed in round bottom 96-well Sarstedt microtiter plates. The plates were pre-incubated for 4 h shaking at 150 rpm, 35 °C in a humid 5 % CO_2_-enriched atmosphere. To each well containing 90 μl of pre-incubated bacteria, 10 μl of one of the antimicrobial concentrations (or PBS) was added, resulting in eight identical rows (one row for each time-point) containing bacteria exposed to 11 different antimicrobial concentrations and one untreated control.

### Estimating bacterial growth rates

The bacterial growth rates (*ψ*) were determined from changes in the density of viable bacteria (CFU/ml) during the first 6 h of the time-kill experiments. The bacterial populations were assumed to grow or die at a constant rate, resulting in an exponential increase or decrease in bacterial density:

$$ N(t)={N}_0\times {e}^{\psi t}. $$

The growth rate was estimated as the coefficient of a linear regression from the logarithm of the colony counts. Maximum likelihood estimation was used to account for the censored data (values below the limit of detection of 100 CFU/ml). For a given antimicrobial, the geometric mean of all measurements at zero hours was used as the first data point. From the growth rate, the bacterial doubling time can be calculated as follows:

$$ {T}_{1/2}=\frac{ \ln (2)}{\psi }. $$

### Pharmacodynamic model

The pharmacodynamic model by Regoes et al. [7] describes the relationship between bacterial growth rates (*ψ*) and the concentration of an antimicrobial (*a*) (Fig. 1):

$$ \psi (a)={\psi}_{\max }-\frac{\left({\psi}_{\max }-{\psi}_{\min}\right){\left(\frac{a}{\mathrm{zMIC}}\right)}^{\kappa }}{{\left(\frac{a}{\mathrm{zMIC}}\right)}^{\kappa }-\frac{\psi_{\min }}{\psi_{\max }}}, $$

where *ψ*_max_ is to the maximal bacterial growth rate in the absence of antimicrobial and *ψ*_min_ is the minimal bacterial growth rate at high concentrations of antimicrobial. zMIC is the pharmacodynamic MIC where the bacterial growth rate is zero (*ψ*(zMIC) = 0). *к* denotes the Hill coefficient, which describes the steepness of the sigmoid relationship between bacterial growth and antimicrobial concentration. For each antimicrobial, four parameters of the pharmacodynamic model were estimated using a self-starter function, implemented in the R software package *drc* [20]. All figures can be reproduced with R code and data from a publicly available GitHub repository [21].

## Results

### Growth of *N. gonorrhoeae*

Growth curves for the five different WHO *N. gonorrhoeae* reference strains (Additional file 1: Figure S1) confirmed that growth was well supported in GW medium. All strains could be grown from a starting inoculum of fewer than 10^3^ CFU/ml and typically had a lag phase of under 4 h. The stationary phase lasted until 36 h for all strains, followed by a steep decline phase. Growth was similar for all strains, with WHO L the only strain that had a slightly longer lag phase (4 h).

### Time-kill curves

**Fig. 2:**
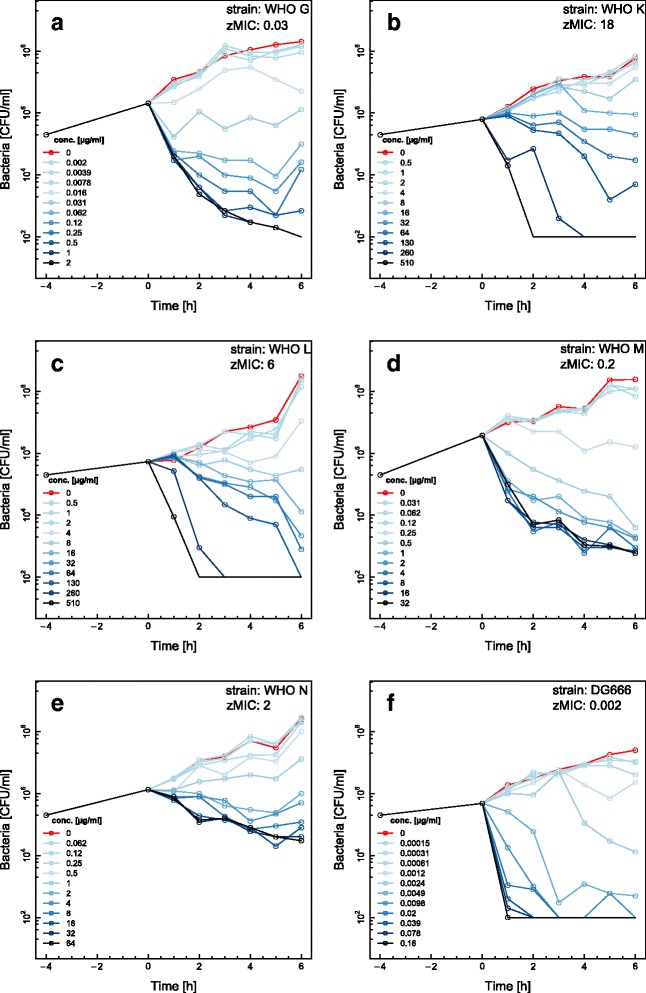
Time-kill curves for ciprofloxacin and six different *Neisseria gonorrhoeae* strains. Time-kill curves for WHO G (**a**), WHO K (**b**), WHO L (**c**), WHO M (**d**), WHO N (**e**) and DG666 (**f**) are shown. Twelve doubling dilutions are plotted, the highest concentration (*black line*) corresponds to 16× MIC as measured with Etest and growth in absence of antimicrobial is drawn in *red*. The antimicrobial was added at timepoint 0 and monitored until 6 h. The limit of detection in the assay was 100 CFU/ml

Time-kill curves for ciprofloxacin using the WHO reference strains WHO G (MIC = 0.125 μg/ml), WHO K (MIC > 32 μg/ml), WHO L (MIC > 32 μg/ml), WHO M (MIC = 2 μg/ml), WHO N (MIC = 4 μg/ml) and DG666 (MIC = 0.008 μg/ml) are shown in Fig. 2. Ciprofloxacin induced a bactericidal effect in all six strains, but the onset of the bactericidal activity was dependent on the concentration of the antimicrobial and differed between strains. All strains with the exception of WHO M and WHO N were killed to below the limit of detection (100 CFU/mL) at the highest antimicrobial concentration (16 fold MIC). The susceptible DG666 strain experienced the most rapid killing during the first hour at high antimicrobial concentrations. For WHO G and WHO M, the bactericidal activity decreased during the 6 h of the assay.

**Fig. 3:**
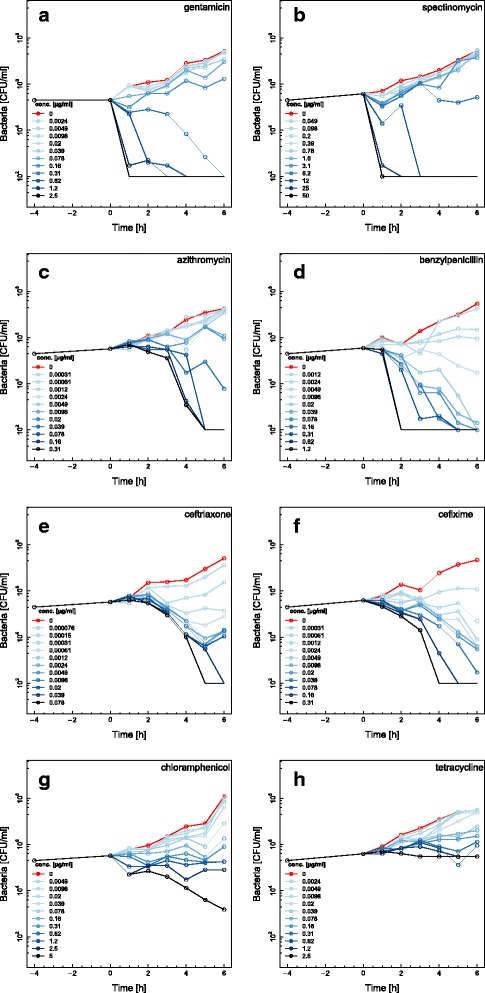
Time-kill curves for the *Neisseria gonorrhoeae* DG666 strain using eight different antimicrobials. The antimicrobial susceptible strain DG666 was exposed to the antimicrobials gentamicin (**a**), spectinomycin (**b**), azithromycin (**c**), benzylpenicillin (**d**), ceftriaxone (**e**), cefixime (**f**), chloramphenicol (**g**) and tetracycline (**h**). Twelve doubling dilutions are plotted, the highest concentration (*black line*) corresponds to 16× MIC as measured with Etest and growth in absence of antimicrobial is drawn in *red*. The antimicrobial was added at timepoint 0 and monitored until 6 h. The limit of detection in the assay was 100 CFU/ml. Data from one of two independent experiments are shown. Additional data are available on GitHub [21]

Time-kill curves for eight additional antimicrobials were also made (spectinomycin, gentamicin, azithromycin, benzylpenicillin, ceftriaxone, cefixime, chloramphenicol and tetracycline) using the highly antimicrobial susceptible DG666 strain (Fig. 3). Similar to the effect of ciprofloxacin (Fig. 2f), gentamicin and spectinomycin exhibited rapid killing during the first 2 h of the assay for concentrations above MIC. Cefixime and ceftriaxone showed little effect from zero to 3 h but the growth rate then decreased rapidly. For benzylpenicillin and azithromycin, at concentrations above MIC, the killing started after 1 h and decreased rapidly at later time points. The time-kill curves for tetracycline and chloramphenicol looked similar with almost no killing of bacteria within the assay time of 4 h. Chloramphenicol showed a weak bactericidal effect at the highest antimicrobial concentration (Fig. 3).

### Pharmacodynamic model

**Fig. 4:**
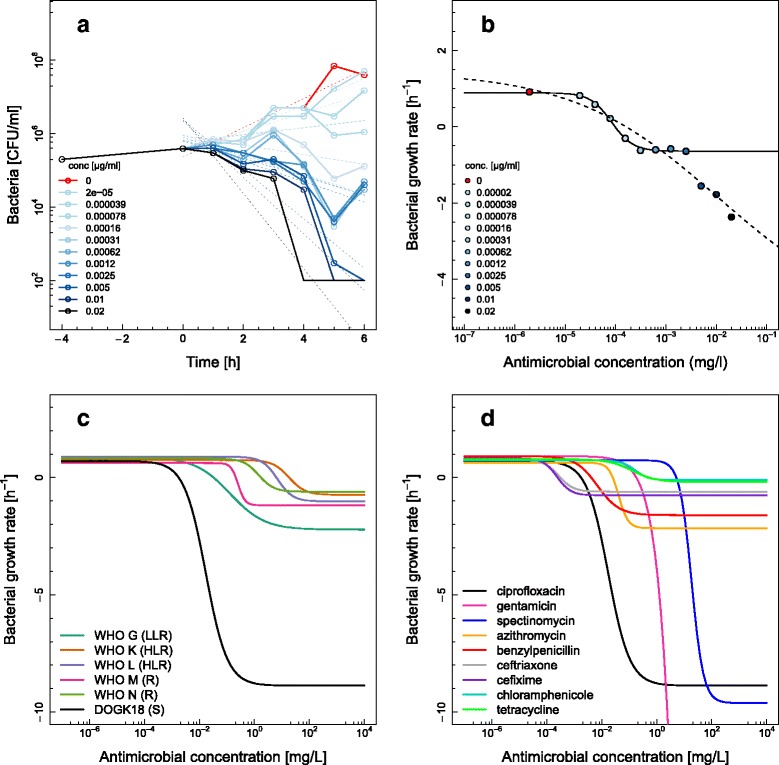
Pharmacodynamic functions for different antimicrobials and *Neisseria gonorrhoeae* strains. **a** Estimating growth rates (cefixime in DG666). Dashed lines represent linear regressions of the logarithm of the colony counts at different antimicrobial concentrations. The coefficient of the linear regression corresponds to the net bacterial growth rate. **b** Fitting the pharmacodynamic function to estimated growth rates (cefixime in DG666). Points correspond to the estimated net bacterial growth rates at different antimicrobial concentrations. The solid line shows the model fit excluding the estimated net bacterial growth rates at very high antimicrobial concentrations. The dashed line indicates the model fit including all data points. The growth rate in absence of antimicrobial is shown in *red* at a concentration that is 10-fold lower than the lowest concentration. **c** Pharmacodynamics functions for ciprofloxacin in six *N. gonorrhoeae* strains (Low Level Resistance (LLR) = WHO G; High Level Resistance (HLR) = WHO K, WHO L; Resistance (R) = WHO M, WHO N; and Susceptible (S) = DG666). **d** Pharmacodynamic functions for nine different antimicrobials in DG666 strain. Note that each curve is based on the arithmetic mean of the estimated parameters from two independent time-kill experiments (as in Table 1)

The bacterial growth rates were estimated from the time-kill curves by fitting a linear regression to the logarithm of the colony counts (Fig. 4a). The pharmacodynamic model was then fitted to the estimated growth rates at different antimicrobial concentrations (Fig. 4b, solid line). In most of the cases exposure to high antimicrobial concentrations (above 16 fold zMIC) resulted in a lower asymptote of the model (*ψ*_min_) and higher concentrations had no additional effect on the growth rate. For chloramphenicol, ceftriaxone, cefixime and benzylpenicillin the growth rate dropped again at very high antimicrobial concentrations. The Hill function could not be fitted appropriately in these cases (Fig. 4b, dashed line), therefore these data points were removed before estimating the parameters of the pharmacodynamic model. However, this phenomenon might indicate distinct biological effects on bacterial growth at different antimicrobial concentrations (see Discussion).

Strains with resistance determinants to ciprofloxacin resulted in significantly changed pharmacodynamic parameters (Fig. 4c and Additional file 1: Table S2). The DG666 strain had a low pharmacodynamic MIC (zMIC) and a low minimal growth rate (*ψ*_min_), indicating the strong bactericidal effect of ciprofloxacin. The five WHO reference strains showed that the ciprofloxacin resistance determinants shifted the zMIC to higher values and resulted in an increase of the minimal growth rate (*ψ*_min_) compared to DG666 strain.

The pharmacodynamic parameters for the nine antimicrobials in the DG666 strain illustrated the different effects that antimicrobials have on the growth of *N. gonorrhoeae* (Fig. 4d and Table 1). The average of the maximal growth rate in the absence of antimicrobials over all experiments was *ψ*_max_ = 0.77 h^−1^ (95 % confidence interval [CI]: 0.71-0.84 h^−1^). This corresponds to a bacterial doubling time of *T*_1/2_ = 54 min (95 % CI: 49–59 min). Ciprofloxacin, spectinomycin and gentamicin induced the strongest bactericidal effect with *ψ*_min_ < −5 h^−1^. Chloramphenicol and tetracycline exhibited almost no killing within the 6 h of the assay (*ψ*_min_ > −0.2 h^−1^). The Hill coefficient *к* ranged between 1.0 and 2.5. The pharmacodynamic parameters were similar for ceftriaxone, cefixime and the bacteriostatic compounds chloramphenicol and tetracycline. Generally, the estimated zMIC agreed well with the MIC measured by Etest (within one doubling dilution) but were lower for benzylpenicillin, ceftriaxone, cefixime and gentamicin.

**Table 1:** Parameter estimates of the pharmacodynamic function for nine antimicrobials in the antimicrobial susceptible *Neisseria gonorrhoeae* strain DG666

Antimicrobial	Antimicrobial class	*к* ^*a*^	*ψ*_min_ (h^−1^)^a^	*ψ*_max_ (h^−1^)^a^	zMIC (μg/ml)^a^	MIC (μg/ml)^b^
Ciprofloxacin	Fluoroquinolone	1.1 ± 0.1	−8.9 ± 2.2	0.7 ± 0.4	0.002 ± 0.0001	<0.004
Gentamicin	Aminoglycoside	1.0 ± 0.2	−106.9 ± 140^c^	0.9 ± 0.07	0.2 ± 0.04	1
Spectinomycin	Aminocyclitol	2.0 ± 0.6	−9.6 ± 1	0.7 ± 0.03	5 ± 0.7	4
Azithromycin	Macrolide	2.5 ± 0.1	−2.2 ± 0.1	0.6 ± 0.05	0.03 ± 0.002	0.19
Benzylpenicillin	β-lactam	1.1 ± 0.1	−1.6 ± 0.6	0.9 ± 0.2	0.004 ± 0.002	0.032
Ceftriaxone	β-lactam	1.6 ± 0.1	−0.6 ± 0.2	0.8 ± 0.07	0.0003 ± 0.0001	<0.002
Cefixime	β-lactam	1.7 ± 0.5	−0.8 ± 0.2	0.8 ± 0.1	0.0002 ± 0.0002	0.016
Chloramphenicol	Chloramphenicol	1.8 ± 0.4	−0.1 ± 0.01	0.7 ± 0.2	0.5 ± 0.1	0.19
Tetracycline	Tetracycline	1.0 ± 0.2	−0.2 ± 0.08	0.8 ± 0.07	0.5 ± 0.3	0.125

^a^Estimates are given as arithmetic means and standard deviations from two independent experiments. Parameter estimates for each individual experiment are given in Additional file 1: Table S1

^b^MIC values measured with Etest in accordance with the manufacturer’s instructions

^c^Parameter below limit of detection

## Discussion

A robust and reliable method to evaluate antimicrobial treatment options in vitro is urgently needed to help tackle the problem of antimicrobial resistant *N. gonorrhoeae*. In this study, a standardised in vitro time-kill curve assay was developed and the resulting data were analysed using a pharmacodynamic model that describes the relationship between the concentration of antimicrobials and the bacterial growth rate [7]. We obtain and compare in vitro pharmacodynamic parameters of antimicrobials in susceptible and resistant strains of the same pathogenic species, opening up avenues into understanding the effects of different resistance determinants on strain phenotype.

The time-kill assay we developed worked well for different *N. gonorrhoeae* strains, including highly resistant isolates. Time-kill assays are usually very laborious but growing the bacteria in 96-microwell plates and using the modified Miles and Misra method [16] for plating made it possible to study 12 antimicrobial concentrations in the same experiment. The assay time was limited to 6 h and growth in the absence of antimicrobials was highly consistent and exponential for all strains during that time. The analysis of time-kill data for the susceptible strain DG666 showed strong bactericidal effects of ciprofloxacin, gentamicin and spectinomycin. Ciprofloxacin is a prime example of a bactericidal antimicrobial, representing the class of topoisomerase II inhibiting fluoroquinolones [22]. The five WHO reference strains used in this study have different ciprofloxacin resistance-conferring mutations in *gyrA*, *parC* and *parE*. This was reflected in an increased pharmacodynamic MIC (zMIC) and a weaker bactericidal effect of ciprofloxacin, showing that even exposure to high concentrations (16 fold MIC) had a limited effect on the growth of these resistant strains.

Spectinomycin and gentamicin both inhibit protein translation [23–25]. Spectinomycin is a well-recognised treatment option for gonorrhoea and resistance is found rarely [26, 27]. Gentamicin is currently the recommended first-line treatment for gonorrhoea in Malawi, where it is used together with doxycycline in the syndromic management of urethritis [28]. This aminoglycoside has been suggested for wider use in the treatment of gonorrhoea recently [29–31] and our time-kill data suggest that further exploration of this treatment option could be rewarding.

The cell wall inhibiting β-lactam antimicrobials are known to have a time-dependent mode of action [32, 33]. Therefore it was not surprising that benzylpenicillin, ceftriaxone and cefixime were characterised by time-dependent, bactericidal killing (−1.6 h^−1^ < *ψ*_min_ < 0.6 h^−1^). Although currently not used for treatment of *N. gonorrhoeae*, chloramphenicol and tetracycline often act as model compounds for bacteriostatic effects [34, 35]. These effects were confirmed by growth rates close to zero at high antimicrobial concentrations (*ψ*_min_). Resistance to tetracycline is widespread [36] and chloramphenicol is relatively toxic and has undesirable side effects [37], so neither of these antimicrobials is currently routinely used for the treatment of gonorrhoea.

The Hill coefficient *k* describes the steepness of the pharmacodynamic curve around the zMIC. Higher values of *k* result in a steeper curve and a more dramatic increase in bacterial killing for increasing antimicrobial concentrations. For low values of *k*, increasing antimicrobial concentrations result in only marginal increases in bacterial killing, suggesting that the time above the zMIC may be a more important correlate for efficacy than a high ratio of maximum concentration to zMIC. Hence, Regoes et al. [7] hypothesised that high and low Hill coefficients are associated with concentration- and time-dependent antimicrobials such as ciprofloxacin and tetracycline, respectively. In our study, we did not find significantly different Hill coefficients for ciprofloxacin and the time-dependent beta-lactams. However time-dependent antimicrobials were clearly associated with higher minimal growth rates (*ψ*_min_) in our data. These results are in line with a review of pharmacodynamic parameters from different organisms and antibiotics. Czock and Keller [38] found a lower maximum kill rate for time-dependent compared to concentration-dependent antimicrobials. The association with time-dependency was less clear for the Hill coefficient and further studies were suggested to confirm a tendency towards higher values in some of the studies [38]. The Hill coefficient (к) might also depend on the genetic background and metabolism of different strains therefore isogenic strains should be studied to systematically explore this parameter.

There are some limitations of the methods used in the present study. First, the rapid bactericidal effects of some antimicrobials occurred immediately after the compound was added resulting in bacterial counts below limit of detection at the first time point. These effects can make it challenging to estimate the minimal growth rate at high concentrations (*ψ*_min_) below values of −10 h^−1^, as observed for gentamicin for example. Second, the estimated bacterial growth rate at high antimicrobial concentrations did not always follow the sigmoidal four parameter model (Fig. 4b). This was the case for the beta-lactams and in one instance for chloramphenicol. Dose-response curves with multiphasic features and more than one inflection point have been observed previously and potentially indicate multiple targets [39]. We therefore hypothesize that these high concentrations induce a biological effect distinct from the primary target and removed them for the scope of this study. Fitting a multiphasic model that could capture this effect would make it difficult to compare the parameters within this study and also to previous studies using the same model [7]. Hence, the pharmacodynamic parameters are only valid within the studied range of antimicrobial concentrations, and benzylpenicillin, cefixime and ceftriaxone could well exhibit stronger bactericidal effects at higher concentrations. Third, the assay time was limited to 6 h to ensure synchronised growth for all strains. Hence, potential regrowth at later time points and post antibiotic effects could not be studied. Fourth, the time-kill curves appeared to level off over time for bactericidal compounds in susceptible strains. Interestingly, this phenomenon might represent a physiological adaptation to those antimicrobials, often described as persister cell formation [40–45]. This non-exponential decline makes it difficult to estimate the growth rate with linear regression. The clinical relevance of persister cells has been demonstrated for chronic infections such as tuberculosis [46] and infections caused by *Staphylococcus aureus* [47]. Homologues to toxin-antitoxin modules involved in persister cell formation have been described also for *N. gonorrhoeae* [48] making it worthwhile considering this phenomenon in future studies. Furthermore, the proposed time-kill method allows the comparative evaluation of antimicrobials against *N. gonorrhoeae* in vitro only and pharmacokinetic effects were not studied.

The in vitro pharmacodynamic parameters can provide relative comparisons across different strains and antimicrobials which can be extremely valuable in preclinical studies. A novel compound can, for example, be categorised and compared to mechanistically well-understood antibiotics [49]. As a next step, the pharmacodynamic properties that are obtained in vitro should be compared to data from clinical PK/PD studies that include additional parameters such as serum concentrations and half-life of the antimicrobial. This will be important to validate whether pharmacodynamic modeling based on in vitro data can be used to predict the outcome of different dosing strategies in vivo. For benzylpenicillin, ceftriaxone and cefixime the time of free antimicrobial above the MIC value should be maximised [50–52], suggesting that multiple dose treatment would be a rational strategy. Fluoroquinolones and aminoglycosides, which act in a concentration dependent and bactericidal manner, should be given as a single high dose [53]. This is typically achieved by maximising the AUC/MIC and peak serum concentration/MIC ratio [54–56]. Our results suggest that this could be the case for ciprofloxacin, gentamicin and spectinomycin, which were found to be strongly bactericidal and concentration dependent. Azithromycin has been described to be bacteriostatic in *Staphylococcus aureus, Streptococcus pneumoniae* and *Haemophilus influenzae* [57] but appears to act bactericidal on *Pseudomonas aeruginosa* [58]. The in vitro pharmacodynamic parameters suggest that there is a continuous gradient from bacteriostatic to bactericidal effects and that azithromycin might fall in between these two categories.

## Conclusions

The present study shows that evaluation of the parameters of a pharmacodynamic model based on in vitro time-kill data can add valuable information beyond that of MIC values for different antimicrobials. The quantitative assessment of pharmacodynamic parameters provides a more detailed picture of antimicrobial-induced effects on *N. gonorrhoeae*. The pharmacodynamic parameters can be applied for the evaluation of new antimicrobials and to study the effects of combining antimicrobials against *N. gonorrhoeae*.

## Additional file

Additional file 1:
**Figure S1.** Growth curves for five WHO reference strains. WHO G (A), WHO K (B), WHO L (C), WHO M (D), WHO N (E). Data from three independent experiments are shown. CFU/ml for each time-point are shown in circles (experiment 1), triangles (experiment 2) and diamonds (experiment 3). A Gompertz growth model was fit to the data from three independent experiments (solid lane, pooled data). Individual fits from each of the experiments are shown as well in dashed lines. Growth rates were estimated in log phase between 2 and 20 h (WHO G = 0.75 [h-1], WHO K = 0.72 [h-1], WHO L = 0.57 [h-1], WHO M = 0.75 [h-1], WHO N = 0.70 [h-1]). The maximal bacterial density was estimated as upper asymptote of the Gompertz model (WHO G = 9.74*109 [CFU/ml], WHO K = 1.32*109 [CFU/ml], WHO L = 6.57*107 [CFU/ml], WHO M = 1.32*109 [CFU/ml], WHO N = 5.32*1011 [CFU/ml]). **Table S1**. Parameter estimates from nine different antimicrobials in DG666 and model based standard errors. **Table S2.** Parameter estimates from ciprofloxacin in five WHO reference strains and model based standard errors. (PDF 489 kb)
