# Supplementary material for: Time-kill curve analysis and pharmacodynamic modelling for in vitro evaluation of antimicrobials against Neisseria gonorrhoeae
Source: BMC Microbiol. 2016 Sep 17;16:216. doi: 10.1186/s12866-016-0838-9 (PMC5027106; doi:10.1186/s12866-016-0838-9)
Supplement: Additional file 1: — Figure S1. Growth curves for five WHO reference strains. WHO G (A), WHO K (B), WHO L (C), WHO M (D), WHO N (E). Data from three independent experiments are shown. CFU/ml for each time-point are shown in circles (experiment 1), triangles (experiment 2) and diamonds (experiment 3). A Gompertz growth model was fit to the data from three independent experiments (solid lane, pooled data). Individual fits from each of the experiments are shown as well in dashed lines. Growth rates were estimated in log phase between 2 and 20 h (WHO G = 0.75 [h-1], WHO K = 0.72 [h-1], WHO L = 0.57 [h-1], WHO M = 0.75 [h-1], WHO N = 0.70 [h-1]). The maximal bacterial density was estimated as upper asymptote of the Gompertz model (WHO G = 9.74*109 [CFU/ml], WHO K = 1.32*109 [CFU/ml], WHO L = 6.57*107 [CFU/ml], WHO M = 1.32*109 [CFU/ml], WHO N = 5.32*1011 [CFU/ml]). Table S1. Parameter estimates from nine different antimicrobials in DG666 and model based standard errors. Table S2. Parameter estimates from ciprofloxacin in five WHO reference strains and model based standard errors. (PDF 489 kb) [file 12866_2016_838_MOESM1_ESM.pdf]

Additional File 1

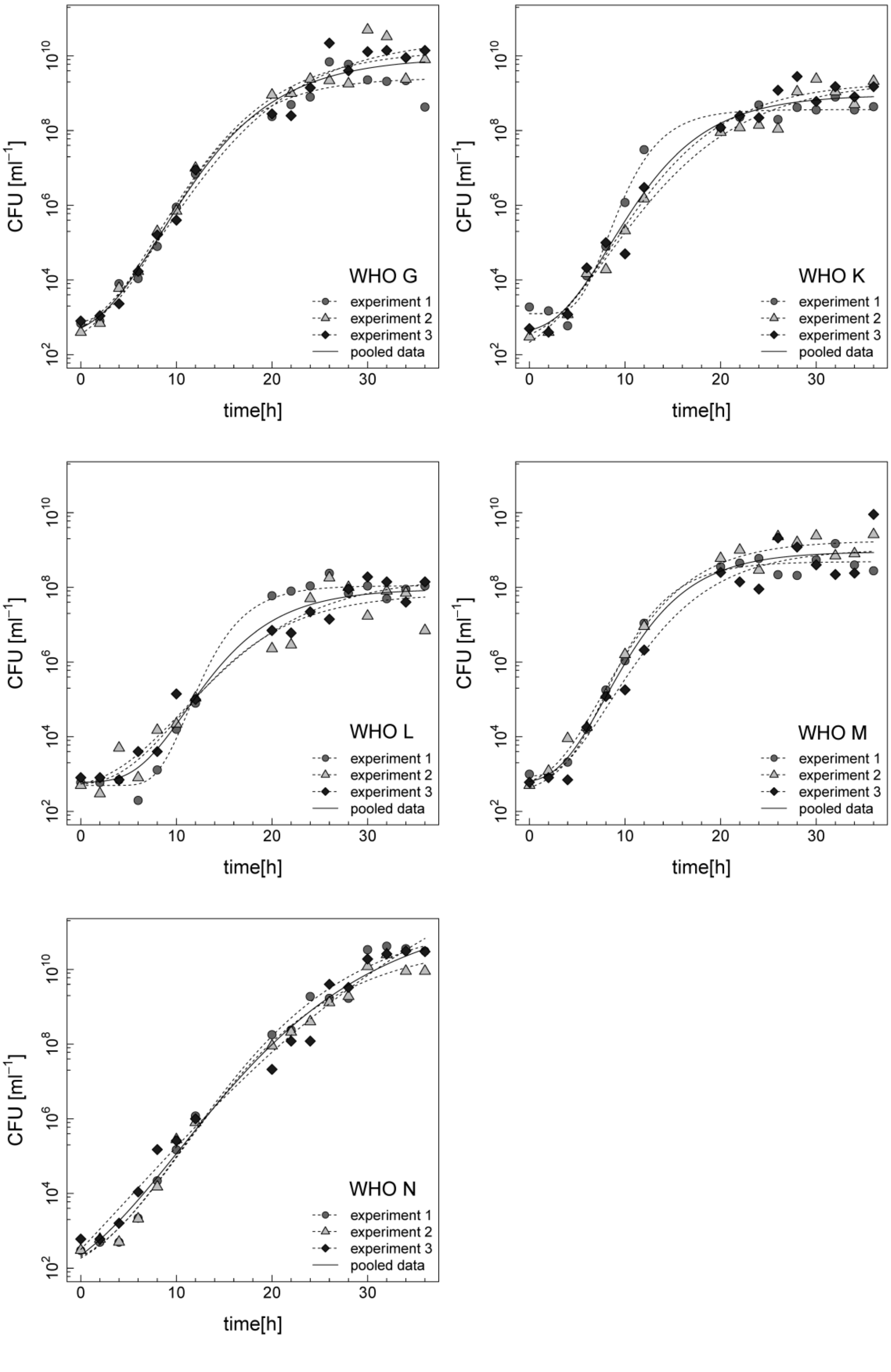

Figure S1. Growth curves for five WHO reference strains. WHO G (A), WHO K (B), WHO L (C), WHO M (D), WHO N (E). Data from three independent experiments are shown. CFU/ml for each time-point are shown in circles (experiment 1), triangles (experiment 2) and diamonds (experiment 3). A Gompertz growth model was fit to the data from three independent experiments (solid lane, pooled data). Individual fits from each of the experiments are shown as well in dashed lines. Growth rates were estimated in log phase between 2-20 hours (WHO G=0.75 [h^{-1}], WHO K=0.72 [h^{-1}], WHO L=0.57 [h^{-1}] , WHO M=0.75 [h^{-1}], WHO N=0.70 [h^{-1}]). The maximal bacterial density was estimated as upper asymptote of the Gompertz model (WHO G=9.74*10^9 [CFU/ml], WHO K=1.32*10^9 [CFU/ml], WHO L=6.57*10^7 [CFU/ml], WHO M=1.32*10^9 [CFU/ml], WHO N=5.32*10^{11} [CFU/ml]).

Table S1: Parameter estimates from nine different antimicrobials in DG666 and model based standard errors.

No	antibiotic	κ	κ SE	ψ_{max} [h^{-1}]	ψ_{max} SE [h^{-1}]	ψ_{min} [h^{-1}]	ψ_{min} SE [h^{-1}]	zMIC [µg/ml]	zMIC SE [µg/ml]
1	azithromycin	2.43	0.81	0.67	0.08	-2.25	0.17	0.0267	0.0050
2	azithromycin	2.64	0.60	0.60	0.06	-2.07	0.11	0.0238	0.0033
3	cefixime	2.07	0.23	0.89	0.04	-0.64	0.03	0.0001	0.0000
4	cefixime	1.42	0.66	0.75	0.17	-0.87	0.10	0.0004	0.0001
5	ceftriaxone	1.69	0.21	0.70	0.05	-0.74	0.03	0.0002	0.0000
6	ceftriaxone	1.58	0.69	0.80	0.08	-0.46	0.09	0.0004	0.0001
7	chloramphenicol	1.54	0.28	0.85	0.03	-0.12	0.04	0.3767	0.1099
8	chloramphenicol	2.04	0.43	0.61	0.02	-0.10	0.03	0.5762	0.1461
9	gentamicin	0.82	0.10	0.86	0.20	-206.80	397.96	0.1522	0.3604
10	gentamicin	1.17	0.28	0.96	0.19	-7.96	1.60	0.2117	0.0474
11	penicillin	1.19	0.29	0.77	0.15	-2.06	0.18	0.0053	0.0013
12	penicillin	1.01	0.19	1.05	0.11	-1.15	0.09	0.0029	0.0005
13	spectinomycin	2.41	0.11	0.76	0.04	-10.30	0.22	5.6452	0.2181
14	spectinomycin	1.61	0.33	0.71	0.22	-8.94	0.96	4.6836	1.2304
15	tetracycline	1.14	0.19	0.72	0.03	-0.25	0.05	0.3259	0.0860
16	tetracycline	0.90	0.17	0.83	0.04	-0.13	0.07	0.7067	0.3721
17	ciprofloxacin	1.04	0.21	0.98	0.25	-7.34	0.70	0.0017	0.0005
18	ciprofloxacin	1.19	0.29	0.43	0.29	-10.40	1.27	0.0018	0.0009

Table S2: Parameter estimates from ciprofloxacin in five WHO reference strains and model based standard errors.

No.	strain	κ	κ SE	ψ_{max}	ψ_{max} SE	ψ_{min}	ψ_{min} SE	zMIC [µg/ml]	zMIC SE [µg/ml]
19	WHOG	0.69	0.14	0.86	0.12	-2.22	0.35	0.0327	0.0094
20	WHOK	1.47	0.29	0.75	0.03	-0.74	0.18	18.3111	3.9561
21	WHOL	1.56	0.27	0.89	0.06	-1.02	0.10	6.3510	0.7475
22	WHOM	3.49	0.77	0.62	0.05	-1.18	0.04	0.2122	0.0142
23	WHON	1.58	0.24	0.81	0.04	-0.60	0.04	1.7405	0.1912
24	DG666	1.19	0.29	0.43	0.29	-10.40	1.27	0.0018	0.0009
